# Mind the gap: representation and outcomes of individuals with schizophrenia or major depressive disorder not eligible for randomized controlled trials

**DOI:** 10.1192/j.eurpsy.2026.10318

**Published:** 2026-07-31

**Authors:** H. Taipale, working group of Aleksi Hamina, Justo Pinzón-Espinosa, Johannes Schneider-Thoma, Joaquim Radua, Orestis Efthimiou, Narcis Cardoner, Christioph U Correll, Brenda W.J.H. Pennix, Andrea Cipriani, Jari Tiihonen, Jurjen J Luykx

**Affiliations:** 1 Department of Clinical Neuroscience, Karolinska Institutet, Stockholm, Sweden; 2 Forensic Psychiatry, Niuvanniemi Hospital, Kuopio, Finland

## Abstract

**Introduction:**

Clinical care guidelines on psychiatric pharmacotherapies are based on evidence derived from randomized controlled trials (RCTs). RCTs typically have strict eligibility criteria, which may lead to situations where evidence on the effectiveness and safety of medications represents only a subset of the real-world population, i.e., unselected patients seen in everyday clinical practice.

**Objectives:**

To quantify the proportion of real-world individuals who would be ineligible for participation in RCTs of schizophrenia or major depressive disorder (MDD), to explore outcomes of eligible versus ineligible individuals, and to compare these rates between schizophrenia and MDD.

**Methods:**

Typical RCT eligibility criteria were applied to real-world populations derived from Finnish and Swedish national registers, in designs mimicking RCTs in terms of duration and inclusion. Proportions of RCT-ineligible individuals were calculated based on any exclusion criteria as well as specific criteria, and risks of outcomes were compared between ineligible versus eligible individuals using Cox regression.

**Results:**

In schizophrenia, 79% of individuals would be ineligible for RCTs, whereas in MDD, the proportion was approximately 34%. These proportions depended heavily on the definition of concomitant somatic conditions as an exclusion criterion. Substance use disorders contributed significantly more to schizophrenia RCT ineligibility than to MDD RCT ineligibility. However, the risk estimates for primary outcomes were considerably larger for MDD than for schizophrenia when comparing ineligible with eligible individuals.

**Conclusions:**

RCTs may face challenges in representing real-world individuals with schizophrenia and MDD. More inclusive eligibility criteria for RCTs are needed to improve the generalizability of trial evidence and the foundation of treatment guidelines.

**Disclosure of Interest:**

H. Taipale Grant / Research support from: Janssen, Speakers bureau of: Gedeon Richter, Janssen, Lundbeck, Otsuka.

